# Registration of Laser Scanning Point Clouds: A Review

**DOI:** 10.3390/s18051641

**Published:** 2018-05-21

**Authors:** Liang Cheng, Song Chen, Xiaoqiang Liu, Hao Xu, Yang Wu, Manchun Li, Yanming Chen

**Affiliations:** 1 Jiangsu Provincial Key Laboratory of Geographic Information Science and Technology, Nanjing University, Nanjing 210093, China; lcheng@nju.edu.cn (L.C.); dz1527007@smail.nju.edu.cn (S.C.); liuxiao_qiang@foxmail.com (X.L.); xuhao_nju@smail.nju.edu.cn (H.X.); wuyang_nju@126.com (Y.W.); limanchun_nju@outlook.com (M.L.); 2 Collaborative Innovation Center for the South Sea Studies, Nanjing University, Nanjing 210093, China; 3 Collaborative Innovation Center of Novel Software Technology and Industrialization, Nanjing University, Nanjing 210093, China; 4 School of Geographic and Oceanographic Sciences, Nanjing University, Nanjing 210093, China

**Keywords:** laser scanning, point clouds, registration, coarse-to-fine strategy, review

## Abstract

The integration of multi-platform, multi-angle, and multi-temporal LiDAR data has become important for geospatial data applications. This paper presents a comprehensive review of LiDAR data registration in the fields of photogrammetry and remote sensing. At present, a coarse-to-fine registration strategy is commonly used for LiDAR point clouds registration. The coarse registration method is first used to achieve a good initial position, based on which registration is then refined utilizing the fine registration method. According to the coarse-to-fine framework, this paper reviews current registration methods and their methodologies, and identifies important differences between them. The lack of standard data and unified evaluation systems is identified as a factor limiting objective comparison of different methods. The paper also describes the most commonly-used point cloud registration error analysis methods. Finally, avenues for future work on LiDAR data registration in terms of applications, data, and technology are discussed. In particular, there is a need to address registration of multi-angle and multi-scale data from various newly available types of LiDAR hardware, which will play an important role in diverse applications such as forest resource surveys, urban energy use, cultural heritage protection, and unmanned vehicles.

## 1. Introduction

Rapid acquisition of spatial information has become important in the development of “geospatial big data”, also facilitating application of these data in social management and scientific research. Data obtained by remote sensing methods are gradually extended from 2D to 3D paradigms and are widely used in professional areas such as geographical monitoring, resource investigation, environmental monitoring, change detection, water surveys, disaster assessment, and other fields. For most current applications, increasing attention is being paid to large-scale, multi-dimensional, comprehensive acquisition of geospatial data. However, it is relatively difficult to meet all these requirements with a single sensor due to limitations of collection range, scanning time, acquisition perspective, and acquisition accuracy. Integration of multi-platform, multi-angle, and multi-temporal remote sensing data is therefore important for geospatial data application.

The registration technique is a core element in integration of multi-platform, multi-angle, and multi-temporal remote sensing data. Early registration techniques mainly focused on 2D image registration. Research in this field began in the 1970s [1], initially for military purposes, but has been gradually extended to remote sensing, medicine, computer vision, and other fields. Image registration methods include the grayness-based and feature-based methods. In general, the feature-based method appears to yield better registration because it considers more contextual image information. Smallest univalue segment assimilating nucleus (SUSAN) [2], scale-invariant feature transform (SIFT) [3], maximally stable extremal regions (MSER) [4], and speeded up robust features (SURF) [5] are widely used operators to extract image features needed for image registration. The development of registration methods has differed in different fields because each field has its own requirements and characteristics. Several researchers have reviewed registration methods and proposed mature image registration frames [6,7,8,9].

As an advanced active remote sensing technology, LiDAR can obtain 3D point clouds of the target object. LiDAR registration is thus 3D rather than 2D. In 1987, the quaternions method was first proposed to estimate transformations between 3D point sets [10]. Subsequently, Besl and McKay proposed a classical iterative closest point (ICP) algorithm for point cloud registration [11]. This method was continuously improved in subsequent research, eventually becoming a comprehensive fine registration method [12]. Early LiDAR point cloud registration was mostly used in industrial fields, with point clouds obtained by a laser scanning system at close distance, and where registration objects were mostly single-target small-scale dense point clouds. Over the last two decades, the LiDAR system has been widely used in earth surface research, such as for forest parameter estimation [13], building reconstruction [14,15], natural disaster monitoring [16], and solar energy potential estimation [17]. LiDAR registration has thus become a key area of research also in the fields of photogrammetry and remote sensing.

There are valuable reviews in the literature of range image or points cloud registration techniques in the fields of computer vision and mobile robotics [12,18,19]. Salvi et al. [18] surveys different pre-2007 techniques for both pair-wise and multi-view range image registration, and provide an overview framework, with techniques ranging from coarse to fine. Tam et al. [19] provide a better understanding of registration from the perspective of data fitting and also consider non-rigid registration. Pomerleau et al. [12] focus on the different ICP variants during the last twenty years as well as their use cases for mobile robotics applications.

Compared with the computer and industrial fields, objects that need to be scanned by the LiDAR system in photogrammetry and remote sensing are mainly larger-scale geospatial features that cover complex and diverse geographical entities and that have distinct spatial stratification. LiDAR point clouds are thus multi-level and have large range, high noise, and small point density, with these being the major factors leading to differences between the point cloud registration algorithms used in photogrammetry and remote sensing and those used in the computer vision field. This paper reviews existing laser scanning point cloud registration methods mainly in photogrammetry and remote sensing, and can thus be regarded as extending the overview of point cloud registration methods to these fields. In order to render the description of registration methods more comprehensive, some registration methods from other fields also are mentioned.

## 2. Brief Presentation of LiDAR Technology and Research

LiDAR has developed rapidly in the past 30 years and the LiDAR sensor can now be mounted on various platforms, including airborne, vehicle, tripod, and satellite platforms. Different platforms have distinct traits and applications, as shown in Table 1.

We performed a search of peer-reviewed journal publications in the Scopus database as of 2016 (using the search statement: TITLE-ABS-KEY (“LiDAR” OR “terrestrial laser scan *” OR “mobile laser scan *” OR “airborne laser scan *” OR “space-based laser scan *”) AND NOT TITLE-ABS-KEY (“aerosol”)), and statistically analyzed the results.

**Figure 1:**
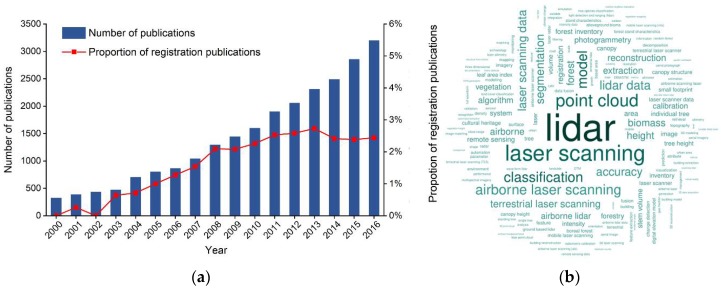
(**a**) Publication statistics; and (**b**) cloud map of high-frequency terms used in LiDAR-related publications (2000–2016). (Source: Scopus Database).

Figure 1a summarizes the number of published articles and reviews discussing LiDAR for each year from 2000 through 2016; the number of publications shows an overall upward trend, indicating the increasing importance of this research. Given the rapid development and significant application of LiDAR, it is useful to summarize current research. A tag cloud map was therefore created based on frequency of occurrence (Figure 1b) [20]. The tag cloud map shows that, from a technology perspective, most papers deal with research and construction of algorithms for LiDAR data, such as classification, segmentation, information extraction, reconstruction, and biomass estimation.

**Figure 2:**
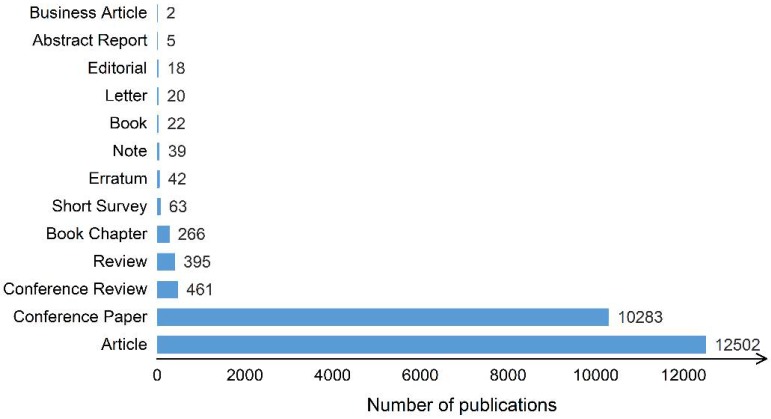
Number of different types of publications on LiDAR (2000–2016). (Source: Scopus database).

Figure 2 shows the proportion of different literature types, with the most common being articles and conference papers; review papers are relatively rare. Available review papers cover forest resource investigations [13,21], land cover classification [22], geological hazard assessment [16,23], building model reconstruction [14,24], road extraction [25], snow depth measurement [26], cryosphere studies [27], and sea ice and ice sheet monitoring [28,29], but no reviews have been published in international peer-reviewed journals discussing registration of laser scanning data in photogrammetry and remote sensing.

We refined our search across publications related to registration (by using the search statement: (TITLE-ABS-KEY(“LiDAR” OR “terrestrial laser scan *” OR “mobile laser scan *” OR “airborne laser scan *” OR “space-based laser scan *”) AND NOT TITLE-ABS-KEY(“aerosol”)) AND (TITLE (“registrat *”) OR KEY(“registrat *”))). Annual variations in percentages of registration-related publications on LiDAR are shown by the red polyline in Figure 1a. This shows that that the proportion of registration-related publications generally increased year-by-year; while researchers continue to expand the range and depth of LiDAR applications, they therefore also continue data-registration research, for the purpose of improving the accuracy and efficiency of LiDAR application by integrating multi-source data.

**Figure 3:**
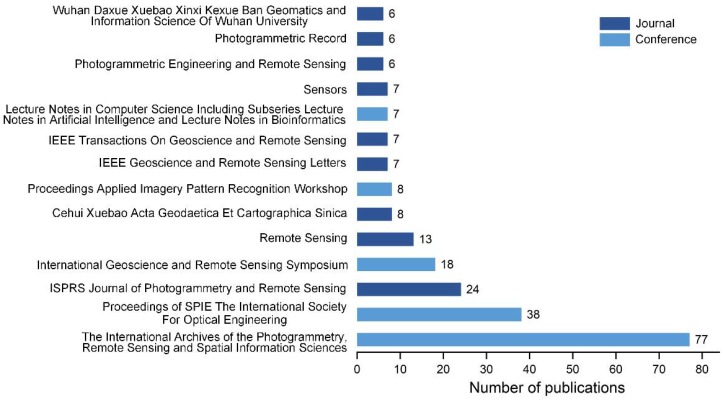
LiDAR registration-related publications in different journals (2000–2016). (Source: Scopus Database)

In total, 501 papers related to LiDAR registration were published in different journals between 2000 and 2016; Figure 3 shows the 14 journals or conferences publishing more than five papers on LiDAR registration. It can be noted that the International Archives of the Photogrammetry, Remote Sensing and Spatial Information Sciences published the most papers related to LiDAR registration, followed by mostly authoritative photogrammetric and remote sensing journals such as the ISPRS Journal of Photogrammetry and Remote Sensing, Remote Sensing, and IEEE Transactions on Geoscience and Remote Sensing. The number of publications in the journal Sensors, which publishes research on the science and technology of sensors and biosensors, is also significant.

In the present paper, we review publications from international peer-reviewed journals on LiDAR registration in the fields of photogrammetry and remote sensing. In order to render the review more comprehensive, we also include important research papers and conference papers that have significant reference value. Section 3 of this paper introduces the classification of LiDAR registration methods and briefly describes the research status of same-platform and different platform LiDAR registration. In Section 4, we discuss in detail current methods used for LiDAR registration based on the principles of methods used. Section 5 briefly introduces current methods used for evaluating the accuracy of LiDAR registration. Section 6 compares different LiDAR registration techniques. Section 7 and Section 8 respectively present discussions and conclusions.

## 3. Classification of LiDAR Registration Methods

Depending on which platform originally generated the point cloud requiring registration, LiDAR point cloud registration can be divided into same-platform registration and registration between different platforms. In earth surface research, there are four main LiDAR systems, i.e., space-based laser scanning (SLS), airborne laser scanning (ALS), mobile laser scanning (MLS), and terrestrial laser scanning (TLS), divided according to the mounted platform (as shown in Table 1). Same-platform registration mainly includes multi-station TLS registration and ALS strip adjustment. For LiDAR registration between different platforms, research mainly focuses on ALS-MLS and ALS-TLS registration.

TLS can obtain accurate location information from global navigation satellite system (GNSS) receivers at the same time the target point clouds are obtained; this location information can be used directly to integrate the different point clouds [30]. However, it is not easy to use GNSS to obtain the exact position of the control point, and even slight instrumental deviations can produce large errors [31]. Furthermore, the spatial accuracy of GNSS in urban areas and forests is limited and prone to lockout, leading to a lack of reliability. Another solution is therefore to set the standard target while simultaneously acquiring the point clouds of the target object, and to then use the standard target to stitch together adjacent-station point clouds. However, in most cases, due to lack of GNSS and standard targets, one must rely only on point clouds themselves for registration.

Due to scanning height and field of view limitations, each flight path for ALS data acquisition can only cover a limited ground width. When large areas must be scanned, many flight paths are required, and a certain degree of overlap between each path must be maintained. Since ALS is an integrated system, there are a number of potential systematic errors for points on the flight paths, including laser ranging errors, sensor mounting errors, and POS and orientation system errors [32,33,34]. In order to eliminate differences between point clouds on flight paths, difference adjustment between different flight paths is necessary. At present, either data-driven methods or sensor system-driven methods can be used to accomplish this adjustment [35]. Data-driven methods use geometric features extracted from the point clouds to calculate the rotation matrix and translation vector [34,36,37]. However, since systematic errors are not linearly distributed, it is difficult to achieve high-precision flight path adjustment using data-driven methods [38], and most researchers opt for the sensor system-driven method, which uses the LiDAR positioning equation as an adjustment model [39,40,41,42]. Both data-driven and sensor system-driven methods require determination of the control unit (point, line, or surface feature), which is used to assess and correct differences between the flight paths [36].

For LiDAR registration between different platforms, research focuses on ALS-MLS and ALS-TLS registration. SLS data are characterized by large spots and sparse distribution and are mainly used for forest resource surveys, and monitoring of sea ice and land ice; integration of SLS data with LiDAR data acquired from other platforms makes no sense. There is therefore no research on registration between SLS and LiDAR data from other platforms.

The data obtained by different LiDAR platforms are heterogeneous in three respects: (1) different perspectives: data collected by SLS and ALS systems are from a top view, while data collected by MLS or TLS systems are from a side view; (2) different spatial resolution: the resolution of ALS data is generally at the meter scale, while MLS and TLS data are at the centimeter scale, with TLS being more precise; (3) different content of focus: ALS data cover general features, while MLS data cover both trajectory sides. Due to the heterogeneity and discreteness of point cloud data, it is very difficult to automatically register two or more point clouds from different platforms. Although there is great potential for automatic registration of point clouds under feature guidance, there are still significant challenges, including how to obtain the conjugate feature which can guide point cloud registration by overcoming heterogeneity, and then how to perform high-quality 3D registration using this conjugate feature.

Point cloud data registration studies frequently apply a coarse-to-fine registration strategy [43,44,45,46,47]. This strategy is not widely adopted for registration between different platforms, but is used for same-platform registration, as described above. In coarse registration, the initial registration parameters for the rigid body transformation of two point clouds are mainly estimated using the feature-based method. In fine registration, the main objective is to achieve maximum overlap of two point clouds, mainly using the iterative approximation method, random sample consensus method, normal distribution transform method, or methods with auxiliary data. We will further describe these methods adopted for coarse-to-fine strategies in Section 4.

## 4. Registration Techniques for LiDAR Data

Based on the coarse-to-fine registration strategy, this section presents an overview of four feature-based coarse registration methods and four fine registration methods. Fine registration methods are iterative approximation methods, random sample consensus methods, normal distributions transform methods, and methods with auxiliary data.

### 4.1. Coarse Registration Methods

The feature-based coarse registration method mainly refers to registration based on point, line and surface features, which possess some invariance over a certain period of time and are widely used for coarse registration [48]. In LiDAR data registration, these features may include building corners, contours, road networks, roof patches, and similar site features [49,50,51]. Since this method uses the feature primitive rather than directly registering the point clouds, identification of appropriate registration primitives is critical for registration accuracy. In practice, discrepancies within LiDAR data arising from use of different platforms (such as different perspectives, different resolutions, and discretization of point cloud data) make it difficult to locate conjugate features of objects to be registered [52], and study of LiDAR data registration methods using conjugate point, line, and surface features remains an area of active research. Here, we classify feature-based methods into four classes: point-based methods, line-based methods, surface-based methods, and others.

#### 4.1.1. Point-Based Methods

Points are most widely used within the feature-based LiDAR registration method [53]. Extraction of feature points is very important in this method and the extraction result directly affects the registration accuracy of point clouds. Compared with natural environments and objects, the artificial objects are often more geometrically regular, and the accuracy of their geometric feature information is relatively high [54]. Building corners, traffic signs, and road signs are therefore commonly-used feature points.

On the other hand, feature points can also be points extracted by using point feature operator, such as point feature histograms [55], spin images of points [56], or scale invariant feature transform features [57]. These feature points extracted using operator are also referred to as keypoints. A good feature operator should possess good noise resistance and be invariant with the rotation and translation of point clouds [3]. There are numerous 3D keypoint operators including local surface patches (LSP) [58], intrinsic shape signatures (ISS) [59], keypoint quality (KPQ) [60], heat kernel signature (HKS) [61], Laplace-Beltrami scale-space (LBSS) [62], Mesh- Difference-of-Gaussians (DOG) [63], and 3D Harris [64]. Tombari et al. [65] and Hänsch et al. [66] survey these 3D operators and compare their performance. As shown in Table 2, point feature, point domain feature, or rotated image feature descriptors are commonly utilized for LiDAR registration.

Registration based on point features still has problems relating to noise sensitivity, low robustness, and large time complexity, and it remains difficult to achieve high precision. Several methodological improvements have recently been proposed in order to address problems of poor computational efficiency and poor robustness, including feature point extraction using the 3D operator for implementation of a point cloud registration algorithm [72,73,74]. In addition, many studies focus on extracting geometric features by constructing the domain topologic information of points and then optimizing the point cloud registration process based on domain features [75]. This approach improves registration accuracy and shows high robustness to noise [66,76]. Aiger et al. proposed a method for implementing point cloud global registration based on 4-point congruent sets (4PCS) of features [77]. This method exploits the fact that the ratio between the lines formed by four coplanar points remains invariant in the process of affine transformation, and does not require calculation of complex geometric characteristics. The method has high efficiency and good anti-noise ability [78], and can achieve automated marker-less registration [79]. Theiler et al. [79] improves 4PCS using 3D keypoints, such as 3D DOG, and 3D Harris keypoints.

It is of note that there are differences in point cloud information obtained using different LiDAR systems to scan the same geographical entities from different perspectives. Even with the same acquisition device, multiple measurements are required to obtain complete target point clouds, especially with TLS equipment, due to obscuration of objects and limited acquisition range. As a result, areas of overlap between top-view LiDAR point clouds and side-view LiDAR point clouds are small, as are multi-station TLS LiDAR point clouds, and extraction of point features is difficult; the point-based registration method therefore cannot be applied in such cases [36].

#### 4.1.2. Line-Based Methods

Lines have stronger geometric topologies and constraints relative to points and permit higher registration accuracy [80,81]. Line features, such as road networks and building contours, are common in large-range 3D point cloud scenes and can be used for LiDAR registration (as shown in Table 3). Buildings are the largest and most important geographical entities in urban spaces, and building contours have been widely used in building model reconstruction and LiDAR point cloud registration [51,76,82]. In addition, roads, which are also important elements of urban space, have been extracted based on their unique linear and regular characteristics [83,84,85] and combined with building contours to achieve registration of point cloud data [46].

Due to the prominence of line features and their ease of extraction, point cloud data registration based on line features has relatively high accuracy and precision. In addition, compared with surface features (described next), the number of line features required during the registration process is relatively small [86]. However, because the completeness and precision of extracted line features are limited, only coarse registration can be achieved.

#### 4.1.3. Surface-Based Methods

Surface features contain more information than line or point features and are less affected by noise. They can therefore be used for automatic registration of LiDAR point clouds. In urban spaces, surfaces are an important element of the ground object structure. LiDAR devices on different platforms can obtain a large amount of ground point cloud data and more precise registration can be achieved by making the best use of these surface features. The extraction accuracy of surface features and their distribution in the point cloud scene directly affect the final registration result. Many researchers have used the least squares method, random sample consensus algorithm, and principal component analysis method for surface fitting, allowing surface features to be obtained in the point cloud scene. The extracted surfaces are mainly ground, roofs, and building facades.

As shown in Table 4, most researchers use the least squares method when performing point cloud registration based on surface features. The method is used to minimize the distance between corresponding surface features of different LiDAR point clouds [89]. When using the least squares method for registration of 3D surfaces, it is necessary to take full account of the randomness of the local surface-normal vector [90]. The accuracy of this method is sufficient for ground deformation monitoring [89]. Some researchers have also implemented point cloud registration by locating conjugate surface features [91,92,93].

Despite the higher accuracy of registration based on surface features, the requirements for point cloud segmentation and the fitting algorithm are high, because surface features must be extracted before registration. In addition, the 3D point cloud scene to be registered must contain numerous surface features [98]; otherwise, it is difficult to guarantee registration accuracy.

#### 4.1.4. Other Feature-Based Methods

Although many studies have used point, line, and surface features to obtain high-accuracy LiDAR point cloud registration, there are still some difficulties relating to large-scale urban 3D point clouds. For example, point-based methods are extremely susceptible to the influence of point density and noise. Most line-based methods are only applicable to buildings when the contours of the building are easy to extract, making these methods difficult to apply to suburbs with fewer buildings. Surface-based methods have high requirements for overlapping areas, and at least three pairs of surfaces should be present in the clouds to be registered [48].

Given these problems, some researchers have considered using a combination of point, line, and surface features to construct a joint transformation model [101], or to find conjugate spatial curves [102], in order to calculate point cloud registration parameters. Alternatively, other registration features, such as circles, spheres, and cylinders, can be used to calculate the registration parameters between different point clouds [103]. However, due to the relatively high extraction requirements, these methods are difficult to extend to general situations and are not widely used [104]. On the other hand, based on the results of feature extraction, if urban 3D point clouds can be classified by semantic analysis [105] and the corresponding relationship between classified surface objects can then be identified, relatively good point cloud registration can be achieved [106].

### 4.2. Fine Registration Methods

#### 4.2.1 Iterative Approximation Methods

In current point cloud registration research, the iterative approximation method mainly refers to the ICP algorithm and its series of improved algorithms. The ICP algorithm is built on the quaternions method, which uses a 4D vector to represent three rotation parameters and one angle parameter [10,107]. The advantage of this method is that it can directly solve rigid body transformation through a rigorous mathematical process, without the need for an initial estimate of location. Besl and McKay first proposed the ICP method for registration of 3D data [11]. This method assumes good estimation of initial location; a number of points are selected from the point set to be registered, and the points corresponding to these points in the reference point set are then identified. The transformation is obtained by minimizing the distance between these pairs of points. The closest point set is then recalculated according to a rigorous solution process and repeated iterations are performed until the objective function value remains constant and the registration result is obtained. This method does not fully consider the effect of noise on accuracy of registration results; however, the effect of noise can be reduced by weighting the least squares distance to improve registration accuracy [108,109,110]. In the computer vision field, in order to speed up the registration process and prevent locally optimized results, several studies register point clouds by calculating feature-substitute point pairs, including invariant features, such as curvature and spherical harmonics [111], surfaces [112], and angular-invariant features [113]. Such ICP-registration methods in computer vision are reviewed in [12,114,115,116].

The development of LiDAR technology has greatly promoted application and development of ICP algorithms for remote sensing and mapping. In the ICP process, it is important to identify the closest point to a known location, with three search strategies used, i.e., point-to-point, point-to-surface, and point-to-projection [90,117,118,119]. However, since the LiDAR device uses discrete laser pulses to measure the distance to a ground target, the target point clouds are practically a dense set of sampling points and do not reflect all details of the target object, especially at the target boundary. Furthermore, due to differences between acquisition devices, angles, and methods, there is no one-to-one correspondence between point sets of LiDAR point clouds from different platforms, and point clouds are easily affected by noise. Registration accuracy is often not ideal and the calculation process is complicated; direct use of the ordinary ICP algorithm therefore often leads to anisotropic and inhomogeneous localization errors. In addition, the ICP algorithm requires that initial location of point clouds should not differ significantly; otherwise, the algorithm will render locally optimal solutions. Many researchers have therefore attempted to improve the ICP algorithm, using strategies that are mainly focused on looking for other registration features, algorithm optimization, and selection of appropriate data-management methods, as shown in Table 5.

Because the ICP algorithm performs point cloud registration based on an iterative process, it is slow at finding corresponding points between two point clouds and is less efficient when registering large-scale, high-density point cloud scenes. However, the ICP concept is used in some registration algorithms for specific surface objects. For example, Bucksch and Khoshelham proposed a registration method based on a tree skeleton line for TLS data from different stations [127]. Optimal conversion parameters were obtained by minimizing the distance between points in input point clouds and the skeleton line in reference point clouds.

#### 4.2.2. Random Sample Consensus Methods

Random sample consensus (RANSAC) methods were proposed by Fischler and Bolles [128], and have been widely used in 2D and 3D data processing; they have also been studied for use in image registration [129,130,131,132]. With the development of LiDAR technology and its application in geography, RANSAC methods have been used for point cloud data preprocessing and segmentation in numerous studies [70,133,134]; their application to point cloud registration has in fact become an important area of research [135]. RANSAC methods involve three steps. First, a number of control points are randomly selected from point cloud data and used to calculate the conversion relationship. Second, the conversion relationship is used to eliminate external points from point cloud data, and the point cloud data registration degree is then calculated. Finally, an iterative transformation is used to find the data set with maximum registration degree, and this is then used to calculate conversion parameters [129]. The process is similar to that of the ICP algorithm, but can avoid iteration over entire point clouds. In combination with the SIFT operator, RANSAC can effectively solve the problem of 3D point cloud data registration without local features, while improving registration efficiency [136,137].

#### 4.2.3. Normal Distribution Transform Methods

Normal Distribution Transform (NDT) methods were first proposed in 2D space [138] and then gradually extended to point cloud data registration in the fields of robotics and photogrammetry [139,140,141].

Applications of NDT are common in mobile robotics, mainly because the robot can obtain the positional relation between two points through the rangefinder when measuring data. With direct initial transformation, the NDT algorithm can be used to quickly and simply achieve fine registration of point clouds. The main idea of this method is to convert point cloud data in a 3D grid into a continuously differentiable probability distribution function. The probability distribution of the samples of each 3D point position measurement in the grid cell is represented by a normal distribution. Subsequently, the probability of normal distribution of two point cloud data sets is optimized using the Hessian Matrix method to achieve point cloud registration [139]. A key process in the NDT algorithm is to build grids for point clouds, but grid size is difficult to determine. The use of different grid sizes to organize point clouds therefore becomes an effective way to establish grids for 3D point clouds [142,143,144].

Since laser scanners used in photogrammetry cannot measure the positional relationship between two points and cannot carry out the initial transformation, to date there has been little research on applications of the NDT algorithm in photogrammetry. Ripperda and Brenner showed that if the laser scanner is set up approximately upright for each scan, LiDAR point clouds can be sliced parallel to the ground and 2D NDT can be applied to the sliced clouds; this was the first application of the NDT algorithm to TLS point cloud registration [145]. However, as the method is still inherently 2D, not extended to 3D space, it presents challenges for wide-range promotion and application. Magnusson et al. developed a 3D NDT algorithm by replacing the 2D space rotation matrix with a 3D space rotation matrix [146].

The NDT algorithm has fast computational speed and high precision. It is especially suitable for processing large-scale and large data-volume point cloud data, but requirements for initial locations of point cloud data remain high. When using the NDT algorithm for point cloud registration, a coarse to fine registration strategy is therefore used, i.e., in the initial registration process, feature-based methods, which do not have strict requirements for initial positioning of point clouds, are used to obtain coarse registration. After the registration result is obtained, the NDT algorithm is used to achieve fine registration. However, there is still a lack of applied research using this approach in large-scale complex geographical environments.

#### 4.2.4. Methods with Auxiliary Data

In the process of acquiring point cloud data, under certain conditions LiDAR equipment can simultaneously obtain target image data and measurement-device location GNSS coordinates. Especially when using TLS to obtain point clouds, a standard target is generally used to quickly stitch multi-station point clouds. Images, GNSS data, and standard targets can therefore effectively assist in registration.

In image-assisted point cloud registration, images are generally used to extract features, including 2D SIFT features [147] and conjugate corner features [35]. These features are described, screened, registered, and mapped to 3D space to find conjugate features [52,148]; the point cloud conversion parameters are then calculated [52,149]. Compared with discrete LiDAR point clouds, the image has rich space-continuous spectral information, so textural features are evident [150], and features based on image extraction have higher reliability and robustness.

GNSS can accurately obtain the coordinates of the ground target. Some LiDAR equipment therefore also records the spatial location of the platform equipment center while acquiring 3D point clouds. Initial global registration of airborne and MLS data can be performed using GNSS information [30,151], but, in complex urban areas, occlusion by buildings can cause GNSS signal lockout, reducing registration accuracy of point clouds [152].

A standard target is widely used in multi-station TLS point cloud stitching, which uses a special standard target as the same name feature for registration. During the scan of objects by LiDAR equipment, these standard targets can be placed in appropriate locations in the scan area. It must be ensured that more than three standard targets are placed between adjacent scanning stations. After obtaining the standard target information, automatic registration can be performed using associated LiDAR registration processing software, such as Cyclone software. In forests, a dense tree canopy can significantly reduce GNSS positioning accuracy and even hinder the acceptance of GNSS signals. Registration of 3D point clouds based on standard target information only has a narrow range of applications due to the challenges posed by complex scanning scenes and difficulties in setting targets.

## 5. Error Analysis Methods

Point cloud registration error analysis is mainly performed to determine the degree of registration between different point clouds in a common area. Because point clouds have discrete characteristics, the accuracy of registration is generally obtained by calculating the offset distance between model point clouds and registration point clouds after transformation. Specifically, the offset distance can be classified as either point-to-point or point-to-surface distance. Quantitative evaluation of LiDAR data registration results on different platforms or the same platform is of great significance for automatic registration theory and algorithmic implementation of 3D laser point cloud data. There may be significant differences in registration results for different scenarios and ranges due to algorithm complexity and differences in applicability. For example, in small-scale digital archiving of cultural heritage sites, registration accuracy should be within the range of centimeters or millimeters. In contrast, in large-scale geographical applications, due to the complexity and diversity of surface morphology, as well as constraints relating to the performance of the acquisition platform, point cloud registration accuracy for registration of a single target is low (generally required within the decimeter level). After performing point cloud registration using a specific method, it is therefore necessary to perform error analysis on the registration results to select the most suitable registration method. There are three main ideas relating to such error analysis:

(1) Comparison with existing registration methods. At present, most registration methods are improved methods developed from existing relatively mature methods. One important process is therefore to compare the results obtained using original and modified methods. This approach is widely used with the ICP algorithm and its improvements. After point cloud registration using the ICP algorithm and improved algorithms, parameters such as average offset distance, maximum offset distance, minimum offset distance, and standard deviation between model point clouds and conversion data of registration point clouds can be obtained, and the performance of the different registration methods can be analyzed. Bae and Lichti employed traditional and improved ICP algorithms for registration of TLS point clouds [153]. They calculated the mean offset distance and standard deviation between points and corresponding surfaces after conversion of registration point clouds and found that the mean offset distance and standard deviation of the traditional ICP algorithm were 2.24 m and 2.55 m, respectively. The improved ICP algorithm had corresponding values of 0.12 m and 1.50 m, respectively. Clearly, registration accuracy was significantly improved with the improved algorithm. In point cloud registration using an ICP-type algorithm, the time efficiency of registration is an important reference index. When analyzing registration results, the time complexity of different methods must therefore also be quantitatively evaluated.
(2) Error analysis based on a reference point. The range of 3D point clouds of a geographical scene obtained by LiDAR is generally large, especially for ALS data. Calculating the offset distance of each registration point will therefore result in large calculation volumes. However, computational complexity can be effectively reduced by selecting reference points from point clouds and calculating offset distances between them. Before data were scanned, Yang et al. manually placed objects in the scanning scene, and object information in LiDAR point clouds from different stations was obtained through TLS equipment [48]. By calculating the offset distance between objects, the registration accuracy of this method was evaluated and compared with that of the method proposed by Dold and Brenner [93].
(3) Error analysis based on a common point. When an ALS system is used to obtain surface 3D point clouds, it is difficult to set reference targets in the scanning scene, and this method is therefore more difficult to apply to analysis of ALS data registration results. Common point clouds, such as the ground points from ALS and MLS, can be selected from LiDAR point clouds and the offset distance between point clouds can then be calculated based on the common point [46,67]. Geographical scenes are unique and complex, and no geographical scenes have exactly the same geographical landscape; the geographical scene at the same location will also change with time. As a result, when validating the scientific meaningfulness and reliability of a proposed method, most researchers focus on specific scenarios and specific objects, rather than natural geographical scenes.

It is difficult to evaluate the advantages and disadvantages of different methods because the evaluation indices used by different authors are not consistent. In order to quantitatively evaluate different methods, it is therefore necessary for authoritative organizations to establish standard data sets for point cloud registration and a set of comprehensive evaluation indices. The International Institute of Photogrammetry has established a set of standard data sets for building 3D reconstructions and surface-cover classifications, which has helped standardize research in these fields.

## 6. Comparison of Different Point Cloud Registration Methods

Geographical scenes contain a large amount of features, especially in urban space, where widely distributed buildings, roads, and transport facilities provide many point, line, and surface features. Such features can be used to quickly achieve registration between different point clouds. Feature-based registration methods are usually applicable for coarse registration, which provides a good initial position for fine registration, effectively reducing computation demands for point cloud registration. A key process in this feature-based approach is feature extraction, which directly affects final registration accuracy. Although the existing feature-based method can achieve good results by searching for conjugate points, lines, or surface features, it is still difficult to use feature-based methods for large-scale LiDAR point cloud registration, because it is difficult to guarantee that extracted features are evenly distributed within the global range. Since point clouds are irregular and discrete, a point-based feature method is more sensitive to the density of point clouds and noise than a line-based or surface-based feature method. At present, most line-feature methods use lines obtained from building point clouds to calculate conversion parameters, but this is relatively difficult in areas with few buildings. The surface-based feature method requires large overlap between different LiDAR point clouds to locate conjugate surface features. In addition, most feature-based registration studies use local features of point clouds, and there is little research on use of global features. Global features can characterize the global characteristics of point clouds, while local features only represent its domain characteristics. The feature-based approach must thus maintain a balance between feature proficiency, method stability, and time efficiency [154].

Most existing feature-based registration methods can only be used to achieve initial registration. In contrast, the iterative approximation method, the random sample consensus method, and the normal distribution transformation model are widely used for fine registration. Because LiDAR point cloud registration using the ICP algorithm occurs through iteration, requirements for the initial position of the point clouds are relatively high. When the initial position of the point clouds is poor, it is difficult to obtain a globally optimal solution. The ICP algorithm also requires high point cloud density; when this is low, registration errors may occur in the search for the closest point. In addition, time complexity is generally high with the ICP algorithm. Selecting an effective feature can help speed up the convergence process and reduce registration time [75]. However, it is still impossible to avoid potential errors during location of the closest point by an effective feature. Such problems have been discussed at the target level [155] and as a local feature of a computational point [153].

An important process in the RANSAC registration method is continuous filtering of registration features during point cloud registration, with the optimal registration feature used to solve conversion parameters. The random sample consensus has been widely used for point cloud registration, and can achieve good registration results even if overlapping areas are small. However, this method requires iterative sampling and calculation of point cloud consistency. The number of iterations has a significant influence on registration speed and accuracy. If the number of iterations is too high, convergence speed is relatively slow, but if the number of iterations is too small, this will lead to poor selection of samples, making it difficult to obtain desired registration results.

Although the NDT algorithm has been widely used for 2D image registration, it is rarely used in 3D LiDAR research. The NDT algorithm does not require knowledge of the corresponding point relationship or extraction of the registration feature from point clouds; consequently, its calculation efficiency and registration precision are higher. However, this method has a significant drawback in that the cost function is not continuous. Since the method first divides the point clouds into grids and then calculates the Gaussian distribution in the grid, the discontinuous cost function cannot guarantee calculation of high precision conversion parameters. If grid size is too large, final registration accuracy is difficult to guarantee. If grid size is too small, the probability distribution function in the body element cannot accurately characterize the surface features. A multi-scale method could effectively solve the problem of determining the grid unit scale. In the image data-assisted point cloud registration process, most registration methods remain feature-based. Use of GNSS and target data depends on known locations of auxiliary data; the principle is simple and easy to implement, but the degree of automation is often not high. In Table 6, we compare these point cloud registration methods.

Additionally, we compare the applications and performances of different point cloud registration methods, as shown in Table 7. These registration methods are classified based on the coarse-to-fine strategies, the applications of these methods are presented by experimental environment, the performances of these methods are compared based on the deviation between reference and registered data, and the information of the experimental environment, experimental data, and deviation of each method is listed according to the paper.

## 7. Further Developments in LiDAR Data Registration

### 7.1. LiDAR Data Registration for Full Space

In order to obtain multi-phase comprehensive 3D spatial information on the Earth’s surface and even the stars, the LiDAR system would need to be mountable on various platforms, and would need to be able to acquire geospatial data at any time needed. The registration of LiDAR data will therefore develop in two directions: micro-refinement and macro-globalization.

In micro-refinement, the development of LiDAR data registration will include indoor/outdoor and ground/underground registration. Registration can be extended from exterior spaces to interior spaces, and even to integrate both spaces. With the maturation of airborne and vehicle-borne LiDAR detection technology, acquisition of 3D geographical information is becoming increasingly common. More recently, with the development of miniaturized and mobile ground LiDAR scanners, fast scans of indoor space have become possible. Global detection of outdoor and indoor 3D space can be achieved through point cloud data registration, which can improve management of small indoor spaces. It will be possible to obtain point cloud data of human living spaces by integrating point cloud data from underground and underwater spaces.

At present, indoor LiDAR data registration is mainly focused on walls, celling, floor [156], or any other key point descriptors [157]. Algorithms tried to improve not only accuracy but also efficiency, which can reach the requirement of building reconstruction and real-time building information acquirement. On the other hand, large scale outdoor LiDAR data registration has attracted lots of attention, so that different scales of LiDAR data sets can work together to achieve applications such as object extraction [22], change detection [158] and scene reconstruction [159]. Although there are some different applications of indoor and outdoor data registration, the combination of indoor and outdoor scenes is the full space of our living circle. The development of indoor/outdoor registration may be located in indoor- outdoor interacted registration, that the full space is not only the target, but also the rules to adjust the result of indoor or outdoor scenes.

Regarding macro-globalization, LiDAR data registration will continue to expand from regional to global space, and even to interplanetary space. Development of point cloud integration technology, as well as the gradual maturation of point cloud data acquisition technology using space-borne stereo imagery, can overcome the large-spot and wide-spacing limitations of space-borne point cloud data. Consequently, registration of satellite point cloud data and airborne, vehicular, and other multi-platform LiDAR data becomes possible. By obtaining high-precision 3D detailed features, we also gain the ability to solve large-scale problems and enhance resource assessment applications, such as for macro-scale forest resource surveys. In addition, with the advancement of planetary exploration, the development of point cloud registration methods that can be used beyond Earth will be helpful for analysis of the spatial distribution of the landscapes of other planets, moons, and asteroids.

### 7.2. New Types of LiDAR Data Registration

At present, the LiDAR systems used in most areas are small-spot discrete systems. Because the signal received by these systems is discrete, single or multiple-pulse echo information, the ability to characterize the vertical structure and physical characteristics of ground surface objects is reduced, restricting application to other fields. With the development of improved LiDAR sensors, a new, full-waveform LiDAR system came into being. Full-waveform LiDAR adds all-digital waveform recording technology to traditional LiDAR, allowing real-time recording of all or part of the laser reflection echo waveform. Mallet and Bretar [160] reviewed four aspects of full-waveform LiDAR: system introduction, processing methods, quantitative analysis, and applications.

Full-waveform LiDAR systems have been mounted in satellites, aircraft, cars, and other platforms. The obtained point cloud information contains all-digital waveform data on ground objects. It is therefore possible to obtain richer quantitative parameters by performing laser signal processing and information mining directly from the waveform. A key element of processing is waveform decomposition, including methods such as Gaussian decomposition, deconvolution, and empirical models [161,162]. Relative to discrete LiDAR point clouds, a full-waveform radar has a stronger ability to describe object structure, and has been widely used in the study of forests and urban areas. Forest area studies include estimation of forest parameters [163,164,165] and modeling of forested areas [166,167]. In urban space, the use of full-waveform LiDAR to study the distribution and structure of urban elements is still uncommon, mainly because multi-pulse signals only form when the laser beam reaches the edge of a building. A small number of studies have focused on the distinction between different materials and the classification of different ground objects [168,169,170].

At present, there are few studies on registration of full-waveform LiDAR; only two related studies were found in the Scopus Database using the search terms “registrat *”, “full-waveform” and “LiDAR”. Although the 3D spatial distribution of full-waveform LiDAR point clouds is similar to that of traditional discrete LiDAR point clouds, further study is needed to allow full use of all-digital waveform data and to achieve more accurate registration. This registration will take full advantage of the characteristics of full-waveform LiDAR point clouds, such as their high density, strong stratification, higher coordinate accuracy, and richer features, further accelerating the application of full-waveform LiDAR to forests and urban space.

The development of new hardware, especially surface scan, line scan, active/passive laser, and femtosecond LiDAR, also present opportunities for LiDAR data registration. The dual-band LiDAR developed in recent years is based on the superposition of near-infrared band detection with the blue-green band, which not only measures 3D information, but can simultaneously obtain water depth and underwater terrain information, overcoming the problem of the incapability of the infrared band to effectively penetrate water. The emerging face array LiDAR has advantages of large grid density and long-distance rapid measurement, overcoming the limitation that LiDAR cannot be used for long-range dynamic target imaging. However, there remain issues with low resolution and a poor signal-to-noise ratio. Development of multi-spectral/hyperspectral LiDAR makes it possible to obtain rich terrain spectral information while detecting 3D surface information.

### 7.3. Technical Development of LiDAR Data Registration

The presently-used methods of point cloud data registration are mainly coarse and fine registration methods. It is likely that these two methods will still be widely used in future and that registration accuracy will continue to improve. With increasing ability to obtain point cloud data from different complex environments, it becomes necessary to test the sensitivity, robustness, and accuracy of different registration methods with data of differing complexity. Furthermore, as point cloud registration is moving in the direction of large-scale scenes, great attention must be focused on the efficiency of point cloud registration for specific engineering applications. Current methods of improving registration efficiency are mainly focused on point cloud storage and indexing; these include the use of octree, quadtree, and R-tree, and the development of registration rules or extraction features. If an effective combination of data mining technology and use of effective information can be integrated, then registration efficiency can be greatly improved. Data mining is an integral part of the knowledge discovery framework, which uses algorithms to search for hidden information in a large volume of data and eventually construct a knowledge model. Data-mining techniques have been applied to variation detection technologies based on remote sensing imagery, object classification, and other research areas. If spatial data-mining technology is applied to point cloud data registration processes, registration efficiency could be greatly improved while increasing registration accuracy. Such studies may become common in future.

## 8. Summary

With improvements in spatial data acquisition capabilities, multi-platform and multi-angle data have attracted more and more attention, and have been widely used in various fields. The application of integrated multi-platform, multi-angle LiDAR data in urban spaces, forest areas, and polar environments has become an important area of research. This paper has presented a comprehensive review of LiDAR data registration from the perspective of photogrammetry and remote sensing, addressing a gap in the literature. LiDAR equipment can be used to obtain a wide range of 3D surface information, but because the geographical environment is relatively complex and subject to rapid changes, point clouds are very susceptible to the influence of noise. Given this, and the discrete characteristics of point clouds, the point cloud registration process is relatively complex, and consequently, most research has adopted a coarse-to-fine registration strategy, achieving good registration outcomes.

In this paper, we focused on this coarse-to-fine strategy and categorized existing registration methods into two major categories, namely coarse and fine LiDAR data registration methods. Based on the feature used, coarse LiDAR data can be classified into point-based, line-based, surface-based, and other methods. For fine registration, iterative approximation methods, random sample consensus methods, normal distributions transform methods, and methods using auxiliary data are extensively used. Classification based on methods allows in-depth understanding of their principles and characteristics, so that an appropriate registration method can be selected based on different data sources; this is also helpful for understanding whether the selected method is universal. Through an effective combination of initial registration and fine registration, high-quality point cloud registration can be achieved. With improvements in LiDAR equipment and expansion of the scope of access, point cloud data scales have increased dramatically. In large-scale data registration, we must consider the data structure of point clouds and storage methods. Especially when using a feature-based approach for point cloud registration, favorable features should be selected to facilitate more efficient registration. It is also necessary to avoid using all LiDAR point clouds as inputs for iterative approximation and random sample consensus methods.

Although LiDAR point cloud registration technology is relatively mature, there is still a need for an objective evaluation system to provide quantitative analysis of different methods and to promote high-quality registration methods. The establishment of standard data sets and the development of evaluation indicators and automatic evaluation platforms by relevant authoritative international organizations in the fields of photogrammetry and remote sensing will promote further research into point cloud registration. To improve point cloud computing efficiency, it is better to register LiDAR point clouds on a large scale and verify the effectiveness and reliability of the registration method, which will help in promoting application of LiDAR data and solving practical problems.

## Figures and Tables

**Table 1:** Comparison of LiDAR systems mounted on different platforms.

Platforms	System Abbreviation	Scanning Perspective	Scanning Range	Point Cloud Density	Application Areas
Airborne	ALS	Top view	Surface shape	Relatively sparse	Terrain mapping, forest surveys, 3D urban areas
Vehicle	MLS	Side view	Stripe shape	Dense	Road mapping, 3D urban areas
Tripod	TLS	Side view	Point shape	Dense	Deformation monitoring, reverse engineering
Satellite	SLS	Top view	Surface shape	Large spot size, low density	Forestry surveys, atmospheric measurements, snow monitoring

**Table 2:** Point-based registration methods for point clouds.

Feature Type	Methods	Test Objects	Data Platform
Point feature	Projection density [49]	Buildings	ALS, TLS
	Movable guidance point registration [67]	Buildings	ALS, TLS
	Geometric shape constraint [48]	Urban scenes	TLS
Point domain feature	Normal vector angle histogram [55]	Urban scenes, Indoor scenes	TLS
	Minimum Euclidean distance of point pairs [68]	Indoor scenes	TLS
Rotated image feature	3D Euclidean distance of point pairs [69]	Urban scenes	TLS
	SIFT operator [70]	Buildings	TLS
	*k*d-tree [71]	Urban scenes	TLS

**Table 3:** Line-based registration methods for point clouds.

Feature Type	Methods	Test Objects	Data Platform
ALS, MLS	Line feature translation, rotation quantity [87]	Urban scenes	ALS, TLS
	Laplacian matrix decomposition [88]	Urban scenes	ALS, TLS
	Point cloud segmentation based on TIN [36]	Urban scenes	ALS
Combination of building contours and road networks	Road networks used for coarse registration, building contours used for fine registration [46]	Urban scenes	

**Table 4:** Surface-based registration methods for point clouds.

Feature Type	Methods	Test Objects	Data Platforms
Least squares surface	Euclidean distance of the corresponding surface [94]	Individual objects	TLS
	Combined with intensity information [95]	Individual objects, indoor scenes	TLS
	3D similarity transformation model [96]	Small plateau	ALS, images
	Stochastic model [97]	Individual objects	TLS
Conjugate surface	Three pairs of conjugate surface features [98]	Urban scene	TLS
	Rodriguez matrix [99]	Buildings	TLS
	2D similarity transformation and simple vertical shift [100]	Buildings	ALS, TLS

**Table 5:** Improved methods based on ICP.

Improvement Strategy	Advantages	Methods
Find other registration features	Effectively reduce noise interference	Variation of geometric curvature of point, variation of normal vector of point and normal vector angle [120]
		Distance from point to tangent plane of closest point in model [121]
		Angle between point and direction of k adjacent points in field [113]
		A point-to-plane method using General Least Squares adjustment model [90]
Optimize registration algorithm	Directly improve algorithm efficiency	Weighted analysis of anisotropic and inhomogeneous registration properties [122]
		Weight matrix in three principal directions calculated by covariance matrix [123]
Select appropriate data management method	Quickly and efficiently store and manage discrete LiDAR point clouds	Octree [124]
		3D *R*-tree [125]
		quad-tree [113]
		*kd*-tree [126]

**Table 6:** Comparison of various point cloud registration methods.

Methods		Main Idea	Advantages	Problems
Feature-based methods		“Feature extraction—feature matching—point clouds registration”, using features to guide point cloud registration	High precision, results are robust and reliable	Requires that target has significant features; extracted feature precision and quality are difficult to guarantee
Iterative approximation methods		Euclidean distances between point clouds are continually reduced by iteration	High precision, and mostly used for fine registration	Requires large overlap area; high requirements for initial position; prone to local optimal solution
Random sample consensus methods		Registration parameters are calculated using smallest sample set	High efficiency, strong anti-noise capability	Number of iterations required for convergence is difficult to determine
Normal distribution transformation methods		Construct body element, generate point cloud distribution model, and determine optimal matching relationship	Efficiency is relatively high; no need for good initial position	Requires point clouds to have large overlapping areas
Methods using auxiliary data	Image-assisted methods	Extract same named feature in image, then use feature matching method	Principle is simple, mostly used in global registration	Image data availability is poor, and it is difficult to ensure quality of extracted feature
	GNSS-assisted methods	GNSS data assisted point cloud coordinate transformation	Principle is simple, mostly used in global registration	Accuracy of GNSS data and signal lockout
	Standard target-assisted methods	Calculate point cloud conversion parameters using standard target information	Principle is simple and easy to operate	Less automation, not suitable for complex scenes

**Table 7:** The applications and performances of different registration methods.

Methods	Experimental Environment		Experimental Data	Deviation (m)
**Point-based methods**	Projection density [49]	Outdoors, urban scene, the campus of Nanjing University, China, covers 1000 × 1000 m^2^	**ALS**: density = 11 points/m^2^; accuracy (*h*) = 0.30 m; accuracy (*v*) = 0.15 m **TLS**: density = 25 points/m^2^	0.50 0.44 (*h*) ^1^ 0.15 (*v*)
	Geometric shape constraint [48]	Outdoors, open park, covers 1450 × 650 × 65 m^3^	**TLS**: density = 442 points/m^2^; 40% overlap	0.068
		Outdoors, uptown, covers 600 × 400 × 30 m^3^	**TLS**: density = 326 points/m^2^; 20% overlap	0.072
		Outdoors, subway station, covers 300 × 450 × 10 m^3^	**TLS**: density = 673 points/m^2^; 50% overlap	0.069
	Movable guidance point [67]	Outdoors, urban building, the campus of Nanjing University, China, covers 400 × 1600 m^2^	**ALS**: density = 1 points/m^2^; accuracy (*h*) = 0.3 m; accuracy (*v*) = 0.2 m **TLS**: at 50 m, density = 100 points/m^2^; accuracy (*h*) = 6 mm; accuracy (*v*) = 4 mm	0.26
	3D distance of point pairs [69]	Outdoors, urban scene, courtyard-like square with manmade objects	**TLS**: angular resolution = 0.12°; 7 scans, each scan contains 2.25 million points	—
		Outdoors, open park area with little structure	**TLS**: angular resolution = 0.12°; 7 scans, each scan contains 2.25 million points	—
	SIFT operator [70]	Outdoors, building object, covers 27 × 12 × 18 m^3^.	**TLS**: angular resolution (*h*) = 0.0015°; angular resolution(*v*) = 0.0015°	0.02
**Line-based methods**	Laplacian matrix decomposition [88]	Outdoors, urban scene, covers 800 × 15,000 m^2^	**ALS**: density = 8 points/m^2^ **TLS**: density = 12 points/m^2^	0.37
		Outdoors, urban scene, covers 11,000 × 12,000 m^2^	**ALS**: density = 5 points/m^2^ **TLS**: density = 20 points/m^2^	0.70
	TIN-based [36]	Outdoors, urban scene	**ALS**: density = 2.24 points/m^2^; accuracy (*h*) = 0.5 m; accuracy(*v*) = 0.15 m	0.007 (*x*) ^2^ 0.004 (*y*) 0.004 (*z*)
	Road networks & building contours [46]	Outdoors, urban scene, Olympic sports center, Nanjing, China, covers 4000 × 4000 m^2^	**ALS**: density = 4 points/m^2^ ; accuracy (h) = 0.30 m; accuracy (v) = 0.15 m **MLS**: 360° scanning cope, surveying range 2–300 m, and point frequency 200,000 points/s	0.68(*h*) 0.41(*v*)
**Surface-based methods**	Rodriguez matrix [99]	Outdoors, building	**TLS**: the scanning interval was roughly 2 cm, 4 stations were positioned about 25 m away from the house.	0.0223 (*x*) 0.0030 (*y*) 0.0206 (*z*)
		Outdoors, substation	**TLS**: the scanning interval was roughly 2 cm, the distance was less than 50 m between the two stations.	
**Other feature-based methods**	conjugate spatial curves [102]	Indoors, No. 159 cave in the Dunhuang Mogao Grottoes	**TLS**: the average span of points was 1 mm, about 35–60% overlap between different scans, and 76 scans consisted of 17.5 million points.	0.003
	fitting of simple objects [103]	Indoors, industrial site, the room is about 8 × 4.5 × 4 m^3^.	**TLS**: 4 scan, each scan consisted of 1 million points.	—
	object detectors [106]	Outdoors, urban scene, streets of New York, Paris, Rome, and San Francisco.	**MLS**: each data set contains 300–500 M points representing 50–100 city blocks covering 2–4 km^2^.	—
**Iterative approximation methods**	point-to-plane [90]	Individual object, the Neil Armstrong statue in Purdue University	**TLS**: 8 scans, positioned at a distance of 5–10 m from the statue	0.0025
**Random sample consensus methods**	SIFT features [136]	Outdoors, urban scene, the data set is acquired at a district in Hanover called Holzmarkt.	**TLS**: angular resolution = 0.12°; a measurement accuracy of 12 mm can be expected.	0.015
	iterative closest projected point [137]	Outdoors, the Ronald McDonald house in Calgary, Canada.	**TLS**: 6 scans, the average overlap is roughly 70%.	—
**Normal distribution transform methods**	2D NDT [145]	Outdoors, urban scene, a street in Hannover	**TLS**: 4 scans, each scan requires about 4 min and yields approximately 2,250,000 scanned points.	0.42
	3D NDT [146]	Outdoors, 3 mine data sets, which are collected in the Kvarntorp mine, south of Örebro in Sweden.	**TLS**: 2 scans from the end section of a tunnel from the same pose, only different resolution.	—
			**TLS**: 2 scans were taken approximately 4 m apart; each scans contain around 27,500 points	—
			**TLS**:65 scans, each scans contain around 95,000 points	—

^1^*h* (*v*) represents the deviation in horizontal (vertical) direction. ^2^ *x* (*y*, *z*) represents the deviation in *x* (*y*, *z*) direction.
